# Femtosecond Laser-Assisted Formation of Hybrid Nanoparticles from Bi-Layer Gold–Silicon Films for Microscale White-Light Source

**DOI:** 10.3390/nano12101756

**Published:** 2022-05-21

**Authors:** Sergei Koromyslov, Eduard Ageev, Ekaterina Ponkratova, Artem Larin, Ivan Shishkin, Denis Danilov, Ivan Mukhin, Sergey Makarov, Dmitry Zuev

**Affiliations:** 1 Department of Physics, ITMO University, 191002 Saint-Petersburg, Russia; sv.korom@gmail.com (S.K.); ekaterina.grachkova@metalab.ifmo.ru (E.P.); artem.larin@metalab.ifmo.ru (A.L.); i.shishkin@metalab.ifmo.ru (I.S.); imukhin@yandex.ru (I.M.); s.makarov@metalab.ifmo.ru (S.M.); d.zuev@metalab.ifmo.ru (D.Z.); 2 Interdisciplinary Resource Center for Nanotechnology, Saint Petersburg State University, 199034 Saint-Petersburg, Russia; danilov1denis@gmail.com; 3 Nanobiotechnology Laboratory, Alferov University, 194021 Saint-Petersburg, Russia

**Keywords:** hybrid nanoparticles, broadband photoluminescence, laser-induced nanoparticles, dewetting, bi-layer gold–silicon films

## Abstract

It is very natural to use silicon as a primary material for microelectronics. However, silicon application in nanophotonics is limited due to the indirect gap of its energy band structure. To improve the silicon emission properties, it can be combined with a plasmonic part. The resulting metal–dielectric (hybrid) nanostructures have shown their excellence compared to simple metallic dielectric nanostructures. Still, in many cases, the fabrication of such structures is time consuming and quite difficult. Here, for the first time, we demonstrate a single-step and lithography-free laser-induced dewetting of bi-layer nanoscale-thickness gold–silicon films supported by a glass substrate to produce hybrid nanoparticles. For obtaining hybrid nanoparticles, we study nonlinear photoluminescence by mapping their optical response and morphology by scanning electron microscopy. This method can be used for the fabrication of arrays of hybrid nanoparticles providing white-light photoluminescence with a good control of their microscopic sizes and position. The developed approach can be useful for a wide range of photonic applications including the all-optical data processing and storage where miniaturization down to micro- and nanoscale together with an efficiency increase is of high demand.

## 1. Introduction

The development of white-light photoluminescence (PL) micro- and nanoscale sources is of great interest to many prospective photonic applications. Silicon is a perfect candidate for building such kinds of light sources due to its compatibility with available well-known technologies. However, silicon has an indirect gap, and the quantum yield of bulk material is very small (on the order of 10${}^{-7}$).

One approach for improving the silicon emission properties is the use of hexagonal polytypes, which can be synthetized by ion implantation into SiO${}_{2}$/Si structures with subsequent annealing [1]. Hybrid nanostructures where silicon is combined with a metallic part are another promising way of enabling the manipulation of optical properties at the micro- and nanoscale [2]. For example, such hybrid nanostructures can be used for radiation control of a single-photon emitter [3]. Moreover, an effective enhancement of the electric field >50 times was experimentally demonstrated in a hybrid nanogap resonator composed of a silicon nanoparticle (Si NP) and gold (Au) flat surface [4]. In turn, this enhancement can boost various other processes and effects [5]: Purcell effect, nonlinear optical processes, absorption enhancement, Raman scattering and directional emission. Namely, for a gold (silver)–silicon combination, several examples could be mentioned. Metal–dielectric nanocavity (Si NP coupled with a thin gold film) was used in [6] for real-time tracing molecular events with a nanoscale thermometry through the monitoring enhanced Raman scattering. In [7], a single silicon nanowire coupled with an Ω-shaped plasmon nanocavity demonstrated a bright visible luminescence due to resonant Purcell effect. Sun et al. [8] reported a hybrid nanoantenna consisting of an inner gold nanodisk and an outer silicon ring cavity with superior fluorescence and directivity enhancement.

The fabrication of hybrid NPs could be realized through different approaches. A eutectic reaction of gold thin films deposited on the Si tip surface was used in [9] and Au rods embedded in the Si substrate were formed in eutectic alloys near the surface after heating; the absorbance of hybrid nanostructures produced in a similar way was calculated in [10] using the finite-difference time domain (FDTD) method. Ruffino et al. [11] used nanosecond laser-induced Au/Si eutectic reaction and dewetting process to produce Au/SiO${}_{2}$ core–shell NPs.

Electron beam lithography (EBL) is the most common approach to provide deterministic nanoscale distributions on metallic or dielectric material. Zuev et al. [12] demonstrated the fabrication of hybrid nanostructures with a magnetic optical response via femtosecond laser melting of asymmetrical metal–dielectric (Au/Si) nanoparticles created by lithographical methods. Furthermore, such asymmetrical hybrid nanostructures can be used to locally color the surface in a tunable way [13], as well as fano resonance [14] and near-field distribution [15].

Spherical NPs can be obtained by laser-induced techniques [16,17] due to ablation in various environments. Makarov et al. [18] used femtosecond laser printing (laser-induced forward transfer, LIFT) to produce a nanoscale white-light source based on a hybrid Si/Au nanoparticle in the form of a Au sponge-like nanostructure filled with Si nanocrystallites [19]. The laser printing of Au/Si core–shell NPs was demonstrated in [20], while the assembly of Si/Au core/shell NPs was developed on the basis of double-beam nanosecond laser ablation in liquid [21]. The same geometry was realized by laser irradiation of a mixed Si-Au colloidal solution obtained by CW laser ablation [22] of corresponding targets in ethanol solution or isopropanol solution containing commercial Si micro-powder and AuCl${}^{4-}$ ions [23]. Si@Au NPs formation was reported in [24], where bare Si and Au NPs were produced by Yb:KGW femtosecond laser ablation in water–ethanol solutions followed by adding 3-aminopropyltrimethoxysilane to Si NPs solution and stirring with aqueous solution of Au NPs. In addition, it should be noted that wet-chemical colloidal synthesis is available to produce various core–shell particles, for example, a Au core with a silica shell [25].

The formation of hybrid NPs requires at least two different materials to be combined with a certain accuracy of component position with respect to another part, resulting in quite complex fabrication steps. So far, EBL together with a direct patterning with a focused ion beam are prime choice to get hybrid structures due to great capabilities for design manipulation. However, both techniques are still quite expensive, unsuitable for the patterning of large areas and limited in the production of spherical geometries. Therefore, laser machining [26] can be used for the fabrication of micro- and nanostructures. Indeed, the femtosecond laser surface pattering of a thin film results in a nanoscale dewetting that could be exploited for the large-scale fabrication of various photonic systems [27]. It was shown both for the creation of functional plasmonic (Au) nanostructures [28] and nanocrystalline Si resonators [29,30]. It is important to note that the production of sub-diffraction limited structures (such as bumps, jets, holes, and periodic structures) in metal films with nanoscale thicknesses using tightly focused laser pulses was demonstrated both for direct [31,32,33] and interfering [34,35,36] femtosecond laser beams.

Here, for the first time, we demonstrate a single-step and lithography-free laser-induced dewetting of bi-layer nanoscale-thickness gold–silicon films supported by a glass substrate to produce hybrid nanoparticles. We study their nonlinear PL by mapping their optical response and morphology by scanning electron microscopy.

## 2. Materials and Methods

### 2.1. Fabrication of Au/Si Bi-Layer Films

Au/Si bi-layers were fabricated using the magnetron sputtering method. The thicknesses of gold films are chosen in accordance with the results [28], which demonstrated the range of thicknesses optimal for single NP formation during femtosecond laser-induced dewetting, while silicon films thicknesses are taken to provide different ratios between gold and silicon (1:10, 1:6, and 1:3). Finally, the following bi-layer Au/Si films are considered: 10/100 nm, 30/180 nm, and 30/90 nm. At first, a silicon layer of corresponding thickness is deposed on the SiO${}_{2}$ substrate of 150 μm thickness. Next, a 10 or 30 nm gold layer is applied over it. The thickness of the layers is controlled by a stylus profilometer Ambios XP-1, and deviation does not exceed 2 nm. The same method of magnetron sputtering is used for preparing single-layered silicon and gold thin films to produce reference NPs for comparison with hybrid ones. The thicknesses of gold and silicon films are 50 nm and 30 nm, correspondingly.

### 2.2. Fabrication of Hybrid Nanoparticles

**Figure 1:**
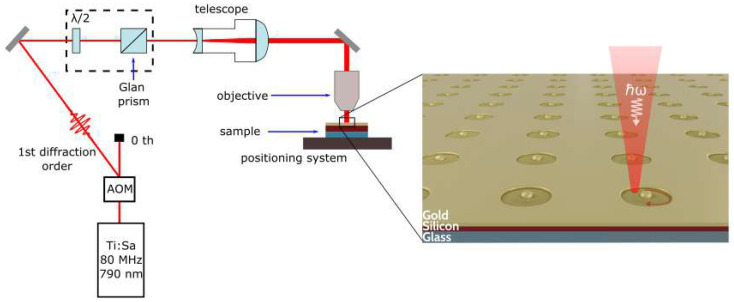
Schematic illustration of experimental setup used for particle fabrication. Inset illustrates a formation of hybrid nanoparticles array under femtosecond laser cutting of patch from a Au/Si bi-layer film on a glass substrate.

Hybrid NPs are obtained from gold/silicon bi-layer films using an experimental setup (Figure 1) based on a commercial femtosecond laser system (femtosecond oscillator TiF-100F, Avesta Poject (Troitsk, Moscow, Russia ) emitting at central wavelength 790 ± 5 nm with a pulse duration of 100 fs at the repetition rate (RR) of 80 MHz. Laser radiation is turned on and off by an acousto-optic modulator (AOM) and goes through a motorized attenuator (dashed rectangle in Figure 1). Laser pulses were tightly focused by a microscope objective (Olympus 40×, NA = 0.75), resulting in the estimated FWHM diameter of beam of d = 1.3 μm in a focal plane. The average laser power ($P_{av}$) was controlled by a power meter (FieldMax II, Coherent, Santa Clara, CA, USA). Pulse energy (*E*) can be calculated as $E=P_{av}/RR$ and the laser fluence (*F*) is $F=E/S=4E/{\left(\pi \cdot d\right)}^{2}$. The samples are placed on a 3D positioning system (air-bearing translating stage driven by brush-less servomotors (ABL1000, Aerotech, Pittsburgh, PA, USA) and exposed during scanning along the circle trajectory at a speed of 5 μm/s. Preliminary experiments were made to find processing conditions (fluence and size of scanning area) providing single NP formation for films of various thicknesses: for 10/100 nm Au/Si film—10 mJ/cm${}^{2}$ and 0.79 μm${}^{2}$, for 30/180 nm—12 mJ/cm${}^{2}$ and 3.14 µm${}^{2}$, and for 30/90 nm—14 mJ/cm${}^{2}$ and 3.14 µm${}^{2}$.

### 2.3. Morphology and Composition Measurements

The geometry characterization and size distribution of produced NPs were analyzed by scanning electron microscopy (SEM). SEM measurements were performed by an Inspect microscope (FEI Company, Hillsboro, OR, USA) with an accelerating voltage of 20 kV in mode of secondary electrons detection. The quantitative composition analyses of NPs was performed by energy-dispersive X-ray spectroscopy (EDX). Analyzed hybrid NPs were located onto carbon tape and investigated by a transmission electron microscope (Libra 200FE, Zeiss, Carl Zeiss AG, Oberkochen, Germany) in STEM mode with 20 nm spatial resolution.

### 2.4. Broadband Photoluminescence Measurements

Nonlinear signal measurements were performed via pumping of NPs by a femtosecond Yb${}^{3+}$ laser TEMA-150 (Avesta Project) which generates pulses with a wavelength of 1050 ± 5 nm, pulse duration of 150 fs, and repetition rate of 80 MHz. After passing through a Glan prism coupled with a half-wave, plate irradiation was attenuated and focused on the sample surface from the substrate side by a 10x microscope objective with NA = 0.26. Excited under pumping, second harmonic generation (SHG) and a PL nonlinear signal were collected from NPs by a 100x objective with NA = 0.7. After that, the signal passed through a FESH1000 filter (Thorlabs, Newton, NJ, USA), which is used for attenuation of the pumping signal and sent to a Horiba LabRam HR spectrometer with a 150 lines/mm diffraction grating and a thermoelectrically cooled CCD (Andor DU 420A-OE 325). The positioning control and recording of nonlinear signal maps of NPs arrays was realized using a piezo stage (AIST-NT), which moves the sample in two perpendicular directions along the sample surface.

## 3. Results and Discussion

**Figure 2:**
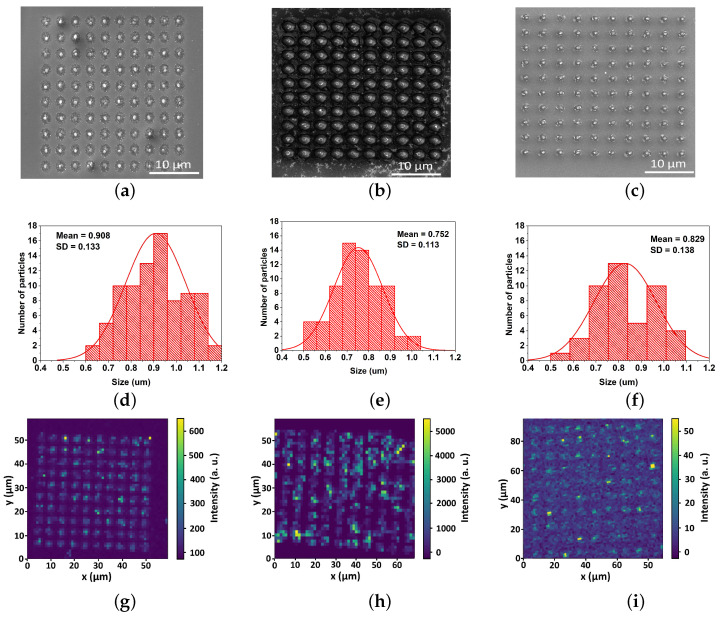
Arrays of hybrid NPs obtained from bi-layer Au/Si films with different thickness (first column—10/100 nm, second column—30/180 nm and third column—30/90 nm): (**a**–**c**) SEM images; (**d**–**f**) size distribution histograms with a mean size (mean) and the standard deviation of the size (SD); (**g**–**i**) PL signal mapping, brighter areas correspond to higher intensity at 600 nm wavelength.

Laser beam scanning of the film surface along a circular path provides the fabrication of nanoparticles arrays for all considered bi-layer films as confirmed by the SEM images in Figure 2a–c. The mean size of NPs varies with the initial bi-layer film thickness in the range of 750–900 nm; the corresponding size distribution histograms are presented in Figure 2d–f. It can be seen that the topography in Figure 2b and the corresponding particle size are different from other two cases. The difference may come from the fact that 30/180 nm film has the biggest thickness compared to another two films (210 nm vs. 110 and 120 nm). Therefore, it may result in different thickness-dependent dewetting morphologies [37,38]. We use strongly focused femtosecond laser irradiation to pattern the bi-layer gold–silicon film with simultaneous heating of the residual film to the dewetting temperatures. It should be noted that film dewetting could start at temperatures lower than the melting point of bulk gold (1337 K) or silicon (1410 K) [39].

Early on [18,19,40], the formation of micro- and nanostructures based on the Au/Si bi-material mixing was demonstrated. Due to laser heating of the bi-layer Au/Si film, it was possible to form liquid nanodroplets of a bi-atomic solution. Furthermore, during a cooling of formed objects at a certain rate, the nucleation and growth processes of silicon nanograins began. The endpoint of the process is the solidification of gold. The final solid-state nanostructure emerges an inclusion of silicon grains in the volume of a spherical gold matrix. Since silicon is insoluble in gold, the metal tries to push out the semiconductor atoms, which are organized into individual nanocrystals. It is assumed that the dewetting leads to the similar formation process as in laser ablation mentioned above. In other words, as a result of the dewetting process, the heated cut patch transforms into a nanoparticle with a complex internal microstructure after cooling.

**Figure 3:**
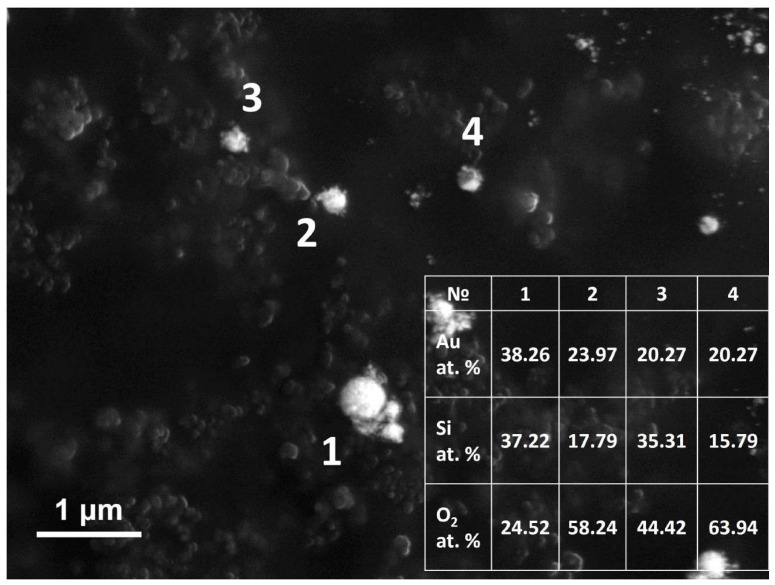
High-angle annular dark field (HAADF) SEM image of hybrid NPs obtained from 30/180 nm Au/Si film and transferred to carbon tape. Legend shows corresponding contents of gold and silicon in atomic percents found by EDX at positions marked by numbers 1–4.

**Figure 4:**
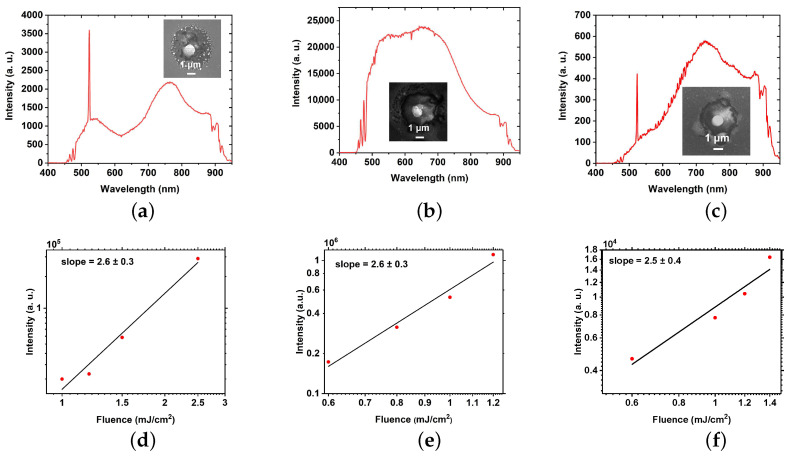

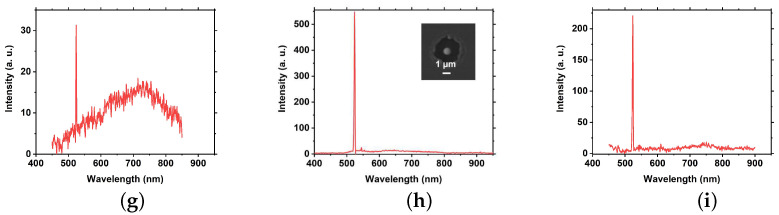
PL measurements of single hybrid NP obtained from bi-layer Au/Si films with different thickness (**a**,**d**) 10/100 nm, (**b**,**e**) 30/180 nm and (**c**,**f**) 30/90 nm: (**a**–**c**) PL spectra; (**d**–**f**) dependencies of broadband PL intensity on pump fluence; PL spectra of initial bi-layer Au/Si (30/180 nm) film (**g**), single gold (**h**) and silicon (**i**) NPs. Insets show corresponding SEM images of single NPs.

The assumption about the formation of a complex internal microstructure is confirmed by several experimental results. First, EDX measurements of single NPs (Figure 3) demonstrate the presence of gold and silicon atoms together with oxygen. Namely, for hybrid NPs fabricated from 30/180 nm Au/Si film, it shows on average 20.3 ± 7.3 at %Au and 25.4 ± 8.4 at %Si. Second, the resulting hybrid NPs can emit broadband photoluminescence upon near-IR excitation (Figure 4, top row). The basis of such a response is the combination of the multiphoton absorption (two and three), re-emission of a high-energy photon (up-conversion), and emission enhancement due to the eigen resonances. It is important to note here that the semiconductor PL spectrum is very sensitive to intrinsic resonances, which have a dominant role in an appearance of resulting PL peaks. Here, we demonstrate experimentally that hybrid NPs of similar shape and size have predominantly different PL spectra. This result can be explained by the existence of a complex internal structure as in [19], where the internal microstructure has characteristic dimensions of the order of the radiation wavelength. Consequently, the resulting PL spectrum is formed from a chaotic internal microstructure, and every NP has its own unique spectrum.

Produced NPs demonstrated both SHG and broadband PL signals (Figure 2g–i and Figure 4a–c) under pumping at 1050 nm. It is important to note that nonlinear PL generated by plasmonic-only nanostructures was observed and studied previously [41,42], demonstrating for the optimized resonant gold NP the rather low quantum yield (<$10^{-5}$ [43] and lower spectral width of PL compared to resonant silicon NPs [44]. Nevertheless, using the same technique, we fabricate gold and silicon NPs from single-layer films to compare their optical response with the produced hybrid NPs and initial bi-layer film. It can be seen clearly (Figure 4g–i) that both reference Au and Si NPs pumped under the same conditions demonstrate moderate SHG and very low PL signals, while the original film gives at least a two orders of magnitude weaker PL signal and an order of magnitude lower SHG signal. To understand such a strong difference, the dependence of PL on pumping fluence is found (Figure 4d–f) for all obtained hybrid NPs. This dependence has similar slopes around 2.6 ± 0.3, manifesting a three-photon absorption-driven NP photoexcitation.

According to our results, NPs fabricated from the bi-layer film of Au (30 nm)/Si (180 nm) thickness demonstrate the strongest PL signal among considered films, as it appears both from PL signal mapping (Figure 2g–i) and PL measurements of single hybrid NPs (Figure 4a–c). It is generally accepted [18,19] that broadband emission in hybrid nanostructures composed of silicon coupled to a plasmonic part essentially comes from the silicon part through multiphoton absorption, while hot electrons and holes generated in gold could be injected into silicon through tunnel action, providing the enhancement of PL efficiency. Therefore, one can assume that this result comes from the highest amount of silicon.

Finally, it should be mentioned that the main limitations factors for reproducibility as well as size control of produced hybrid NPs are laser source long-term stability during the fabrication process along with the proper adjustment of setup and uniformity of initial bi-layer film.

## 4. Conclusions

To the best of our knowledge, for the first time, we demonstrate a single-step and lithography-free laser-induced formation of hybrid nanoparticles from bi-layer nanoscale-thickness gold–silicon film caused by the simultaneous cutting and dewetting of the initial film. The presented fabrication method makes it possible to produce large nanostructures (up to 1 μm in diameter) with the possibility of their precise positioning. Produced hybrid nanoparticles demonstrate the appearance of intense broadband photoluminescence extended from visible to near-IR caused by nonlinear three-photon absorption process. Such hybrid nanoparticles can serve as a nanoscale white-light source and therefore are of great interest for development light-emitting devices in various nanophotonic applications.

## Data Availability

Not applicable.
